# Supplementary material for: The genome sequence of the planthopper, Conomelus anceps (Germar, 1821)
Source: Wellcome Open Res. 2024 Oct 17;9:605. [Version 1] doi: 10.12688/wellcomeopenres.23104.1 (PMC11555367; doi:10.12688/wellcomeopenres.23104.1)
Supplement: Peer review history

# Reviewer report: Reviewer response for version 1

**DOI:** 10.21956/wellcomeopenres.25441.r109489

**Published:** 2024-11-11

**Authors:** Lim Li

**Affiliations:** 1 Universiti Sains Malaysia, George Town, Malaysia

General comment:

 This manuscript provides a thorough and well-structured account of the genome sequencing of a planthopper species. The authors have successfully generated a high-quality genome assembly with extensive coverage and resolution. The methods are detailed, allowing for reproducibility.

 Specific comments:

 Figure 1: The insect photograph should be taken under a dissecting microscope for clarity and include an appropriate scale bar.

 Genome Sequence Report Section: There is an incomplete sentence that should be revised for clarity: "The total count of gaps in the scaffolds is."

Are sufficient details of methods and materials provided to allow replication by others?

Yes

Is the rationale for creating the dataset(s) clearly described?

Yes

Are the datasets clearly presented in a useable and accessible format?

Yes

Are the protocols appropriate and is the work technically sound?

Yes

Reviewer Expertise:

Molecular biology, entomology, plant pathology

I confirm that I have read this submission and believe that I have an appropriate level of expertise to confirm that it is of an acceptable scientific standard.

# Reviewer report: Reviewer response for version 1

**DOI:** 10.21956/wellcomeopenres.25441.r106332

**Published:** 2024-11-02

**Authors:** Qianquan Chen

**Affiliations:** 1 Guizhou Normal University, Guiyang, Guizhou, China

*Conomelus anceps*, a species of planthopper, is a globally distributed herbivorous pest. It has two wing forms: long-winged and short-winged. Authors got a chromosomally complete genome sequence for *C. anceps*. The genome information can contribute to the study of the *C. anceps*. I advise authors to use a new photo which can clearly show the morphological characteristics of *C. anceps*.

Are sufficient details of methods and materials provided to allow replication by others?

Yes

Is the rationale for creating the dataset(s) clearly described?

Yes

Are the datasets clearly presented in a useable and accessible format?

Yes

Are the protocols appropriate and is the work technically sound?

Yes

Reviewer Expertise:

Eco-genomics

I confirm that I have read this submission and believe that I have an appropriate level of expertise to confirm that it is of an acceptable scientific standard.
